# Achieving High Sensitivity and Linearity in Resistive Flexible Sensors Using FeNWs@Graphene as Conductive Fillers

**DOI:** 10.3390/nano15211673

**Published:** 2025-11-04

**Authors:** Lei Cui, Zhengfeng Cao, Chuan Chen, Qiang Zhang, Fangyuan Chang, Yan Xiao, Yiyang Tang, Lining Wu, Xiangyu Ge

**Affiliations:** 1 State Key Laboratory of Advanced Power Transmission Technology, China Electric Power Research Institute Co., Ltd., Beijing 102209, China; cuilei@geiri.sgcc.com.cn (L.C.); chenchuan1@epri.sgcc.com.cn (C.C.); qzhang628@126.com (Q.Z.); changfangyuan@epri.sgcc.com.cn (F.C.); xiaoyan@epri.sgcc.com.cn (Y.X.); 2 School of Integrated Circuits, Chongqing University of Posts and Telecommunications, Chongqing 400065, China; tyiyang1998@126.com; 3 School of Energy Power and Mechanical Engineering, North China Electric Power University, Beijing 102206, China; 4 School of Mechanical Engineering, Beijing Institute of Technology, Beijing 100081, China; gexy@bit.edu.cn

**Keywords:** FeNWs@Graphene, flexible sensors, conductive filler, sensitivity

## Abstract

There is a critical demand for flexible resistive sensors that combine high sensitivity with a wide linear range, fast response speed, and excellent long-term stability. This study presents the development of a high-performance resistive flexible sensor utilizing graphene-coated iron nanowires (Fe NWs@Graphene) as conductive fillers within a polyurethane sponge (PUS) substrate. The sensor was constructed with a sandwich-like structure, consisting of Fe NWs@Graphene-impregnated PUS as the sensing layer, encapsulated by polydimethylsiloxane (PDMS) for protection. The Fe NWs were synthesized via a chemical reduction process employing an external magnetic field. Subsequent chemical vapor deposition enabled uniform graphene coating on the surface of Fe NWs. Systematic performance assessments demonstrated that the Fe NWs@Graphene flexible sensor exhibits a gauge factor (GF) of 14.5 within a 0–100% strain range, representing a 71% improvement over previously reported Fe NW-based strain sensors, along with excellent linearity (R^2^ = 0.994). The sensor also showed rapid response times (113 ms for loading and 97 ms for unloading) and outstanding cyclic stability over 3000 stretching cycles at 50% strain. These enhancements are attributed to the synergistic effects between Fe NWs and graphene: the graphene shell effectively protects the Fe NW core against oxidation, thereby improving stability, and facilitates efficient charge transport, while the Fe NWs serve as bridging agents that improve both mechanical integrity and electrical percolation. In addition, application tests simulating human motion detection confirmed the sensor’s ability to accurately capture muscle-induced strain signals with high repeatability. The results underscore the feasibility of Fe NWs@Graphene as conductive fillers for high-sensitivity, wide-range, and stable flexible sensors, highlighting the potential in wearable electronics and human–machine interaction systems.

## 1. Introduction

The demand for high-performance flexible sensing technology is growing, driven by rapid advances in artificial intelligence, healthcare, wearable devices, and intelligent robotics [1,2,3,4,5,6,7]. Resistive flexible sensors stand out among many flexible sensing solutions due to their simple working principle, low preparation cost, fast response speed, and convenient signal acquisition, demonstrating great application potential and market prospects [8,9,10]. The working principle of resistive flexible sensors relies on variations in electrical resistance, which occur when an external mechanical stimulus alters the material’s conductive network. When the sensor is stretched, bent, or compressed, the structure and distribution of the internal conductive path change, resulting in a change in resistance value [10,11,12,13,14]. This change has a certain corresponding relationship with the external stimulus, and the physical signal can be accurately measured by detecting the resistance change.

As a critical component, the conductive filler significantly influences the functionality of resistive flexible sensors [15,16,17]. The most widely adopted materials for this purpose are metal-based and carbon-based nanomaterials [18,19,20,21,22,23]. Among these, carbon nanotubes (CNTs) and graphene stand out due to their superior electrical and mechanical properties, making them extensively utilized in the field [10,19,23]. However, their high compliance often makes it challenging to maintain a linear response across a wide strain range, thereby limiting practical applications [10,24]. In contrast, metal nanowires (NWs), characterized by a high aspect ratio and elastic modulus, can form stable conductive networks and demonstrate excellent linearity. Metal nanowires such as Ag NWs and Cu NWs have been widely used as conductive fillers in the preparation of resistive flexible sensors [25,26,27,28,29]. However, flexible sensors using Ag or Cu NWs frequently fail to integrate high sensitivity with a wide strain range. This paradox is likely due to their extreme conductivity, which can be detrimental to achieving optimal sensing performance [10,30].

Fe NWs possesses a favorable level of conductivity, thus positioning them as a potential alternative to overcome the shortcomings associated with Ag and Cu NWs for high-performance sensing applications [30,31]. In addition, Fe NWs has unique magnetic control construction characteristics, which can be controlled by an external magnetic field to form microstructures with higher aspect ratios, thus facilitating the construction of more efficient and stable conductive pathways [32]. However, flexible sensors based on Fe NWs suffer from poor long-term stability due to the susceptibility of the nanowires to environmental oxidation [30,31,32].

Considering the excellent mechanical and electrical properties of graphene, it was proposed to composite Fe NWs with graphene to prepare graphene coated Fe NWs (Fe NWs@Graphene) as conductive fillers. This strategy utilizes graphene to passivate the surface of Fe NWs, effectively delaying the oxidation process [30,33,34,35,36]. On the other hand, it leverages the excellent conductivity of Fe NWs to achieve bridging effects, thereby broadening the strain response range of graphene [31]. Meanwhile, compared with the previous studies [10,30,31], this work employs the chemical vapor deposition method to directly prepare graphene coatings on the surface of Fe NWs, which can achieve better passivation effect and long-term stability. Polyurethane sponge (PUS) is formed by the cross-linking reaction between polyols and isocyanates, which creates a three-dimensional network, followed by the action of a blowing agent to generate a 3D porous structure. This material is characterized by a high porosity of 80–95% and a pore size distribution ranging from several tens to hundreds of micrometers. Its mechanical properties, which are highly dependent on this microstructure, exhibit excellent elasticity and flexibility, typically with an elongation at break exceeding 100%. Owing to the structural features and mechanical properties, the PUS was chosen as the flexible matrix. This structure aids in achieving filler dispersion uniformity and establishing a stable conductive pathway with minimal filler content [37,38,39]. Based on the above design, a sandwich structured resistive flexible sensor was constructed by loading Fe NWs@Graphene onto the PUS skeleton to form a continuous conductive pathway, and then encapsulating it with polydimethylsiloxane (PDMS). The strain sensing performances of the fabricated sensors, including tensile characteristics, hysteresis behavior, response speed, and long-term stability, were systematically studied and the relevant mechanisms was also discussed in depth.

## 2. Materials and Methods

### 2.1. Materials

FeSO_4_·7H_2_O, NaBH_4_, anhydrous ethanol, PUS (porosity: 90%, pore size: 200 µm) and PDMS were obtained from the Sinopharm Chemical Reagent Co., Ltd., Shanghai, China. CH_4_, H_2_ and Ar gas were purchased from Beijing AP Baif Gases Industry Co., Ltd., Beijing, China. A pair of permanent magnets (150 mm × 100 mm × 25 mm) generated the required magnetic field.

### 2.2. Fabrication of Fe NWs

**Figure 1:**
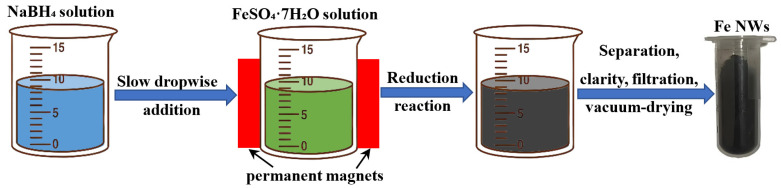
Schematic diagram of the preparation of Fe NWs.

As shown in Figure 1, the magnetic field-assisted aqueous-phase chemical reduction method was employed to synthesize Fe NWs by reducing Fe^2+^ ions with BH_4_^−^ anions. According to the previous study [32], the specific procedures were as follows: First, appropriate amounts of FeSO_4_·7H_2_O and NaBH_4_ powders were separately dissolved in deionized water to prepare aqueous solutions with concentrations of 0.1 mol/L and 1.5 mol/L, respectively. Subsequently, permanent magnets were placed on both sides of the container holding the FeSO_4_·7H_2_O solution to provide an external magnetic field. Next, the NaBH_4_ aqueous solution was slowly dripped into the container, during which a large number of bubbles were observed. The slow addition of the NaBH_4_ solution was continued until no more bubbles were generated. Then, the product was separated and collected using NdFeB permanent magnets, followed by washing with ethanol, filtration, and vacuum-drying, ultimately obtaining the Fe NWs. Meanwhile, the same method was also employed to synthesize the Fe NWs without external magnetic field as contrastive sample.

### 2.3. Fabrication of Fe NWs@Graphene

**Figure 2:**
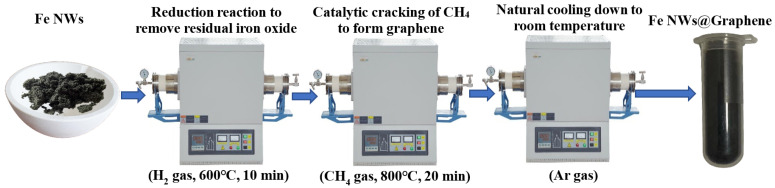
Schematic diagram of the preparation of Fe NWs@Graphene.

As shown in Figure 2, the as-prepared Fe NWs in a crucible were placed into a horizontal tube furnace, where they were treated under a H_2_ atmosphere (80 sccm) at 600 °C for 10 min. This step aims to remove a small amount of iron oxide residues in the Fe NWs through a reduction reaction. Subsequently, the H_2_ gas was stopped and switched to CH_4_ gas (100 sccm), while the temperature was increased to 800 °C and maintained for 20 min. After the reaction, the CH_4_ gas flow was halted and replaced with Ar gas as a protective atmosphere at a flow rate of 80 sccm, followed by natural cooling down to room temperature. Finally, Fe NWs@Graphene were obtained. During the preparation process, a Ni-based catalyst was introduced to lower the decomposition temperature of CH_4_ gas, thereby reducing the adverse effects of high temperature on the structure of the Fe NWs.

### 2.4. Fabrication of the Fe NWs@Graphene Flexible Sensor

**Figure 3:**
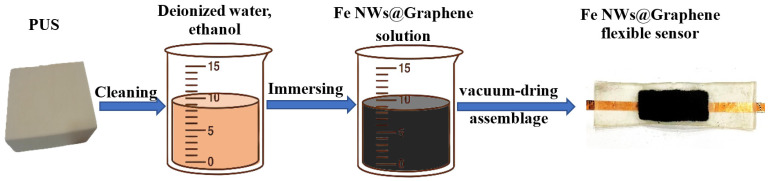
Schematic diagram of the preparation process of Fe NWs@Graphene flexible sensor.

The flexible sensor exhibits a typical sandwich-structure, with upper and lower transparent protective layers made of PDMS, and a middle sensitive layer composed of Fe NWs@Graphene PUS. As shown in Figure 3, the sensitive layer of Fe NWs@Graphene PUS was prepared using a simple dip-coating method. First, a porous PUS substrate (25 mm × 10 mm × 1 mm) was ultrasonically cleaned in deionized water and ethanol, and subsequently oven-dried at 80 °C. An ethanol solution of Fe NWs@Graphene (0.08 mol/L) was prepared, followed by immersion and 30-min ultrasonication of the PUS substrate to ensure uniform dispersion. Finally, the sample was vacuum-dried at 80 °C for 10 min, thereby obtaining the Fe NWs@Graphene PUS flexible sensor. Meanwhile, the Fe NWs PUS flexible sensor was also prepared by the same method to be used as the contrastive sample.

### 2.5. Characterization

The morphology and crystal structure of the Fe NWs were examined using SEM and XRD, respectively. The Fe NWs@Graphene composite was further analyzed by SEM coupled with EDS (SEM-EDS) to reveal its surface topography and elemental composition. Additional structural and chemical properties were investigated through XRD and Raman spectroscopy. The resistivity of the prepared Fe NWs and Fe NWs@Graphene was measured by four-point probe method. The chemical state of Fe element of the Fe NWs and Fe NWs@Graphene before and after cycling were detected by an X-ray photoelectron spectroscopy (XPS). To evaluate the electromechanical performance, cyclic tensile tests were conducted with a universal testing machine. The sensor was clamped at both ends and subjected to repeated stretching up to 100% strain at a crosshead speed of 10 mm/s. Throughout the test, resistance changes were recorded in real time using a source measure unit (SMU), while displacement and electrical signals were simultaneously collected and processed via computer for strain-response analysis.

## 3. Results and Discussion

### 3.1. Characterization of Fe NWs

**Figure 4:**
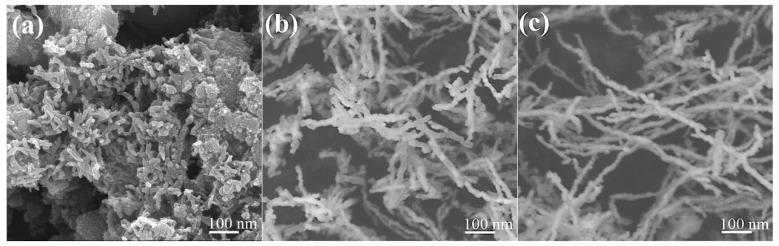
Surface morphologies of Fe NWs: (**a**) reaction without external magnetic field for 2 min, reaction with external magnetic field for (**b**) 2 min and (**c**) 4 min.

Figure 4 shows the SEM images of the surface morphology of the as-prepared Fe NWs. A comparison between Figure 4a and Figure 4b,c reveals a significant effect of the external magnetic field on the morphology of the Fe NWs. As shown in Figure 4a, the Fe NWs prepared without an external magnetic field are relatively short in size and accompanied by a large amount of irregular mixtures. Figure 4b displays that Fe NWs with a relatively high linear structure were successfully synthesized after a 2-min reaction under an external magnetic field. The Fe NWs reacted for 2 min had average diameters and lengths of ~21 nm and ~210 nm, respectively. Figure 4c further illustrates the morphology of the sample after a 4-min reaction under the applied magnetic field, indicating that as the reaction time increases, so does the length of the resulting Fe NWs: thus, the Fe NWs produced under these conditions exhibited average diameters and lengths of ~24 nm and ~470 nm, therefore resulting in a two-fold increase in the NW aspect ratio, from ~10 to ~20. The reasons for the differences in the morphology of the Fe NWs under the different reaction conditions can be explained as follows: The synthesis of Fe NWs proceeds via reduction, nucleation, growth, and assembly in a heterogeneous mixture of NaBH_4_ and FeSO_4_·7H_2_O solution. Fe^2+^ is reduced by BH_4_^−^ to generate elemental Fe, and due to the inherent magnetism of Fe, it undergoes directional migration under the influence of an external magnetic field [32]. Therefore, the formation of Fe NWs is facilitated under magnetic field assistance, and as the reaction time prolongs, the Fe NWs continue to grow, thereby exhibiting a large aspect ratio.

**Figure 5:**
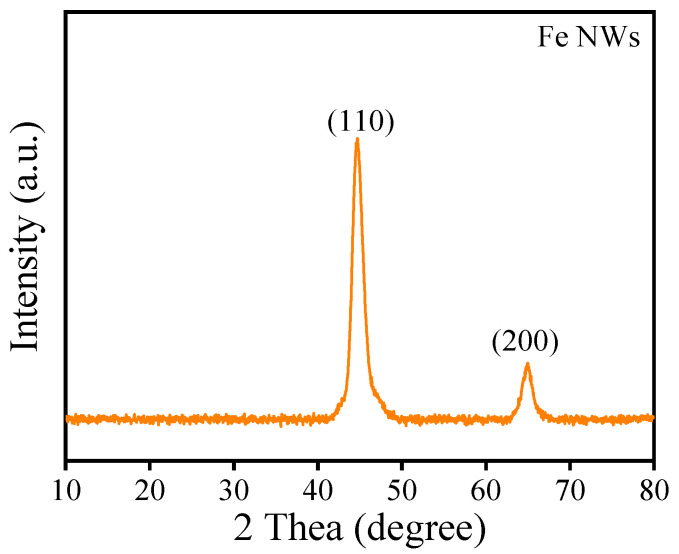
XRD pattern of the Fe NWs.

Considering that the Fe NWs prepared with a reaction time of 4 min under an external magnetic field exhibit a high aspect ratio, the Fe NWs synthesized under this condition were used in all subsequent experiments. As shown in Figure 5, the X-ray diffractogram of the Fe NWs reveals two prominent peaks corresponding to the (110) and (200) planes, consistent with the standard pattern for body-centered cubic (bcc) α-Fe [32]. Furthermore, no additional peaks from impurities are detected in the pattern, indicating that the prepared the Fe NWs are well-crystallized and of high purity [32].

### 3.2. Characterization of Fe NWs@Graphene

**Figure 6:**
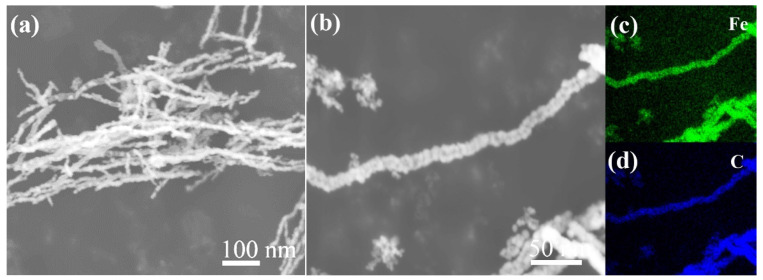
Surface morphologies and mapping distribution images of the typical elements of Fe NWs@Graphene: (**a**) low magnification and (**b**) high magnification SEM images, (**c**) Fe element and (**d**) C element mapping distribution images.

Figure 6 displays the morphology and typical elemental mapping distribution results of the prepared Fe NWs@Graphene. As can be seen from the Figure 6a,b, after being coated with graphene, the Fe NWs@Graphene still maintain the typical one-dimensional nanowire morphology and high aspect ratio characteristics. Figure 6c,d reveal that carbon elements are uniformly and densely distributed on the surface of the Fe NWs, indicating the successful construction of a structurally uniform and dense graphene coating.

The resistivity of the prepared Fe NWs and Fe NWs@Graphene was also measured by four-point probe method. The test results indicate that Fe-NWs exhibit a relatively high resistivity of ~5.74 × 10^−5^ Ω·m, which is attributed to the susceptibility to oxidation in air. In contrast, FeNWs@graphene demonstrate a lower resistivity of ~3.28 × 10^−6^ Ω·m, resulting from the synergistic effects of the passivation and excellent conductivity of the graphene.

**Figure 7:**
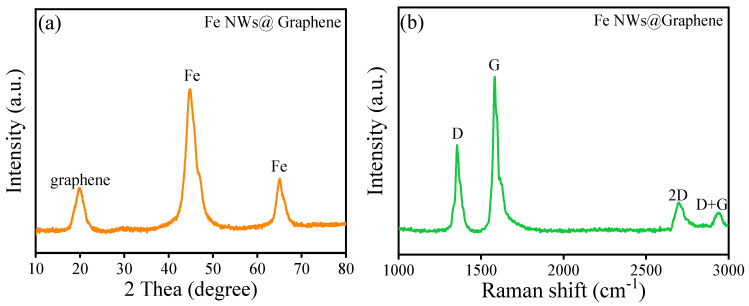
Crystal phase structure and composition of the Fe NWs@Graphene: (**a**) XRD spectra and (**b**) Raman spectra.

Figure 7 presents the XRD and Raman spectra of the Fe NWs@Graphene, employed for characterizing its crystal phase structure and composition. As shown in Figure 7a, the Fe NWs@Graphene still retain two typical diffraction peaks, attributed to the α-Fe phase [32]. Additionally, a diffraction peak corresponding to graphene appears at 2θ = 19.86° [40,41,42]. According to Bragg’s law, the interlayer spacing of the graphene is calculated to be approximately 4.47 Å (0.447 nm). As shown in Figure 7b, the Fe NWs@Graphene exhibit distinct D and G peaks, which originate from lattice defects of carbon atoms and the in-plane stretching vibrations of sp^2^ hybridized carbon, respectively, further confirming the presence of graphene. Meanwhile, the appearance of the 2D peak and the D+G peak can also be observed, indicating that the graphene has a multilayer structure [40,41,42]. The XRD and Raman results further indicate the successful preparation of Fe NWs@Graphene materials.

### 3.3. Sensing Performances of Fe NWs@Graphene PUS Sensor

**Figure 8:**
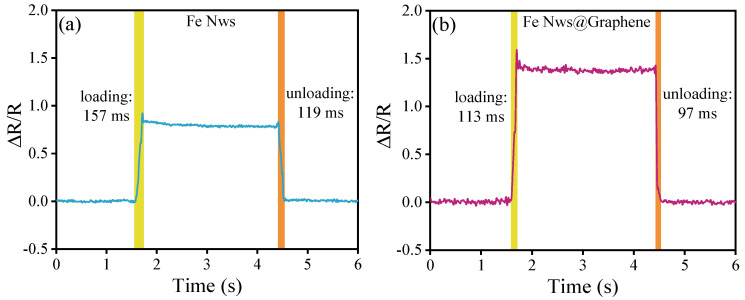
Response speed during loading and unloading under 10% strain: (**a**) Fe NWs flexible sensor, (**b**) Fe NWs@Graphene flexible sensor.

Response time is a critical factor for assessing the performance of flexible sensors. Enhancing this property can be achieved by modifying the composition of the sensing material or refining the sensor’s structural architecture. As shown in Figure 8, the Fe-NWs flexible sensor exhibits a fast and reversible sensing response, and the measured data indicates the loading and unloading times are 157 ms and 119 ms, respectively. In comparison, the Fe NWs@Graphene sensor exhibits shorter response times, with loading and unloading times of only 113 ms and 97 ms, respectively. It is worth noting that the sensing units of both sensors are based on PUS and fabricated using the same preparation process. Therefore, the difference in response time is mainly attributed to the distinct conductive fillers. The above results indicate that coating the surface of Fe NWs with graphene can effectively enhance the response speed of the flexible sensor.

**Figure 9:**
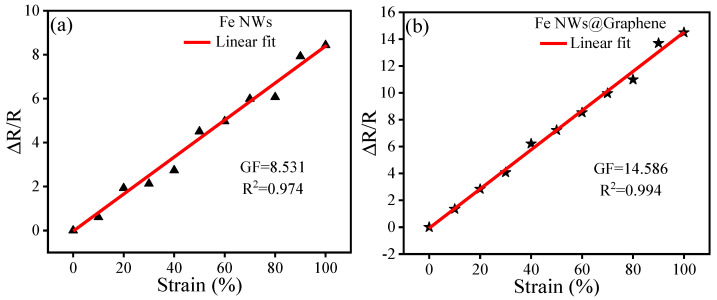
Linearity and sensitivity of the flexible sensors: (**a**) GF and R^2^ values of the Fe NWs flexible sensor, (**b**) GF and R^2^ values of the Fe NWs@Graphene flexible sensor.

Figure 9 presents the tensile testing results of Fe NWs and Fe NWs@Graphene flexible sensors across strain levels from 0% to 100%. The relative resistance change, ΔR/R_0_, is calculated as (R − R_0_)/R_0_, where R_0_ represents the initial resistance and R is the resistance under strain. The gauge factor (GF) is given by GF = (ΔR/R_0_)/ε, with ε denoting the applied strain. As illustrated in Figure 9, the GF of the Fe NWs sensor is approximately 8.5, whereas the Fe NWs@Graphene composite sensor exhibits a significantly higher GF of 14.5—an improvement of about 70.98% over the former. This indicates that the Fe NWs@Graphene flexible sensor exhibits better sensitivity. Furthermore, both types of flexible sensors demonstrate a linear relationship between tensile strain and relative resistance change within the 0–100% stretching range, with the NWs@Graphene flexible sensor showing better linearity (R^2^ = 0.994).

The sensing mechanism of resistive flexible sensors relies on the formation of an internal conductive network through point contacts. Under tensile strain, the conductive materials move along the direction of the external force. At low strain levels, the change in resistance mainly results from the displacement of contact points. At higher strain levels, some contact points separate, leading to the formation of microcracks. These cracks reduce the number of conductive paths, thereby increasing resistance [30,31,32,37]. Compared to the Fe NWs, Fe NWs@Graphene not only possess higher electrical conductivity but also the graphene shell can help prevent oxidation of the Fe NWs, which significantly reduces the initial resistance of the sensor. As a result, during stretching, the relative resistance change becomes more pronounced, thereby leading to a higher GF and better linearity. This combination of properties contributes to the excellent sensing performances of the Fe NWs@Graphene flexible sensor.

**Figure 10:**
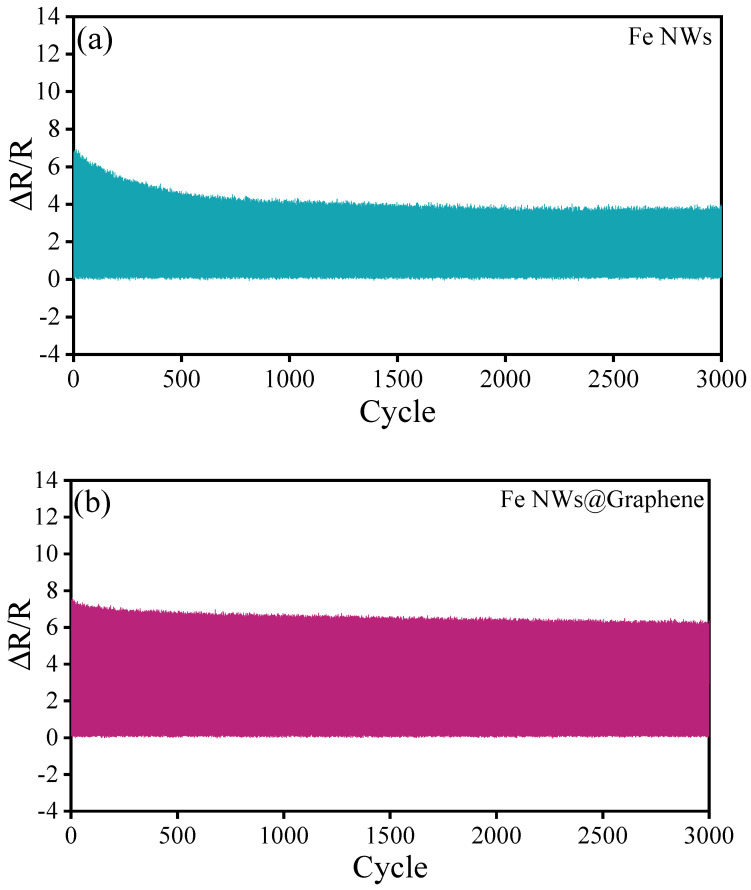
Repeatability under stretching 3000 cycles at 50% strain: (**a**) Fe NWs flexible sensor, (**b**) Fe NWs@Graphene flexible sensor.

Stability is also one of the key indicators for evaluating sensor performance. The fabricated flexible sensors were subjected to repeated testing to evaluate their durability. Figure 10 illustrates the relative resistance change of the Fe NWs and Fe NWs@Graphene sensors after 3000 cyclic tests at 50% strain. As seen in Figure 10a, the Fe NWs sensor demonstrates inferior stability, with noticeable detachment of the sensitive material occurring after about 500 cycles. This degradation results in a gradual reduction in resistance variation and a significant loss of sensitivity. In contrast, as shown in Figure 10b, the Fe NWs@Graphene flexible sensor demonstrates excellent stability. Except for a slight decrease in resistance change at the initial stage, the relative change in resistance remains relatively stable throughout the test and maintains a high value. The results indicate that the Fe NWs@Graphene flexible sensor not only performs well in terms of sensitivity but also possesses outstanding repeatability and stability.

**Figure 11:**
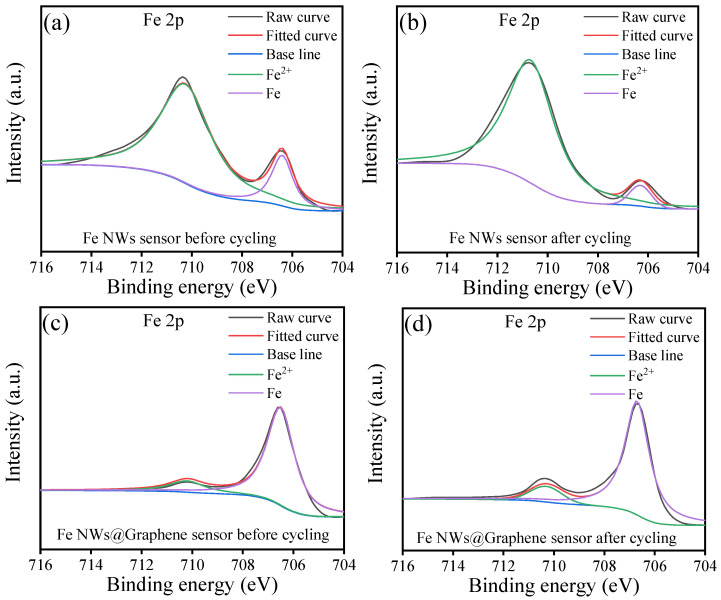
XPS spectra of Fe 2p: (**a**) Fe NWs flexible sensor before cycling, (**b**) Fe NWs flexible sensor after cycling, (**c**) Fe NWs@Graphene flexible sensor before cycling, (**d**) Fe NWs@Graphene flexible sensor after cycling.

As shown in Figure 11, it can be observed that due to the susceptibility of Fe to oxidation in air, the Fe 2p XPS spectra of the Fe NWs flexible sensors exhibit strong Fe^2+^ peaks both before and after cycling, while the metallic Fe peaks are relatively weak (Figure 11a,b). In sharp contrast, the Fe NWs @Graphene flexible sensors display strong metallic Fe peaks with weak Fe^2+^ peaks before and after cycling (Figure 11c,d). This result provides direct evidence that depositing graphene coating on the surface of Fe NWs effectively enhances the oxidation resistance. Furthermore, it can be noted that the Fe^2+^ peaks of both the Fe NWs and Fe NWs@Graphene sensors become more stronger after cycling, indicating an increased degree of oxidation. This suggests that the performance of the flexible sensors may experience certain degradation after cycling, which is consistent with the results presented in Figure 10.

The sensing performance, linearity, and stability of the Fe NWs@Graphene flexible sensor have been systematically evaluated through tensile testing and repeatability tests. Table 1 further provides a performance comparison between the sensor proposed in this study and other reported sensors. It can be observed that the Fe NWs@Graphene flexible sensor demonstrates excellent overall performances, particularly outstanding in terms of Stretchability, linearity, and response time. These results indicate that the Fe NWs@Graphene flexible sensor possesses remarkable sensing capabilities and stability, showing promising application potential.

### 3.4. Application of Fe NWs@Graphene Flexible Sensor

**Figure 12:**
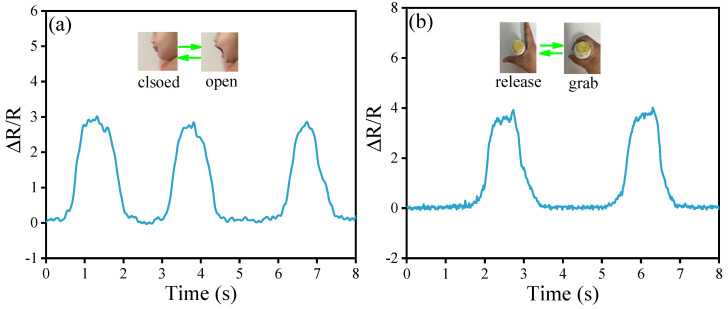
Response curves of Fe NWs@Graphene flexible sensors under the simulated applications: (**a**) Resistance variation signal when opening mouth; (**b**) Resistance variation signal when grabbing the bottle.

The above experimental results indicate that the as-prepared Fe NWs@Graphene flexible sensors exhibit good sensitivity, a wide detection range, fast response speed, excellent stability, and repeatability, demonstrating significant application potential. To further validate its practical performance, simulated application tests were conducted on the sensor. As shown in Figure 12a,b, the FeNWs@Graphene flexible sensors were attached to the corner of the mouth and the index finger of the volunteer while maintaining mouth closure and finger extension. When the volunteer performed mouth-opening and bottle-grabbing actions, the FeNWs@Graphene flexible sensors correspondingly experienced strain, leading to a change in the resistance signals. Figure 12a,b show that the FeNWs@Graphene flexible sensor successfully captured signals generated by muscle stretching during these movements, with good repeatability. These findings fully demonstrate the excellent sensing performance of the Fe NWs@Graphene flexible sensor with a 3D porous structure, highlighting its broad application prospects in the fields of wearable devices and human motion detection.

## 4. Conclusions

In this work, a novel resistive flexible sensor based on Fe NWs@Graphene was successfully fabricated and systematically characterized. The Fe NWs, synthesized under an external magnetic field, displayed high aspect ratios and well-crystallized structures. Subsequent chemical vapor deposition enabled uniform graphene coating on the surface of Fe NWs. The sensor has a sandwich-like structure consisting of Fe NWs@Graphene PUS sensing layer and PDMS protective layers. Fe NWs provides mechanical integrity and electrical percolation, while the graphene layer protects the Fe NWs core against ambient oxidation and introduces additional π–π pathways and facilitates low contact resistance. Detailed characterizations reveal that the Fe NWs@Graphene sensor delivers a GF value of 14.5 across the 0–100% strain range with excellent linearity (R^2^ = 0.994), surpassing some previously reported metal- or carbon-based sensors. The response speed is sufficiently fast. More importantly, the sensor withstands 3000 loading cycles at 50% strain without observable performance decay, whereas the Fe NWs sensor degrades after only 500 cycles. The enhanced durability is attributed to the graphene shell that mitigates oxidative fracture of Fe and stabilizes the percolation network. Meanwhile, practical demonstrations further confirm the capability of Fe NWs@Graphene flexible sensor to detect the skin strain, highlighting its potential in wearable devices and human motion detection. This study confirms that Fe NWs@Graphene is a highly promising conductive filler material for developing high-performance flexible sensors.

## Figures and Tables

**Table 1:** Comparison of stretchability, sensitivity, linearity, and response time of Fe NWs@Graphene flexible sensors with other reported sensors.

Sensors	Stretchability	GF (max)	Linear Range	Response Time	Reference
Fe NWs/CNT/PUS	110%	7.4	0–110%	130 ms (10%)	[10]
Carbon sponge/PDMS	60%	130.49	0–30%, 30–50%, 50–60%	50 ms (0.5%)	[20]
Fe NWs/Graphene/PEDOT:PSS	100%	10.65	10–100%	260 ms (80%)	[30]
rGO/Fe NWs/CMC	10%	112.44	0–10%	128 ms (6%)	[31]
ZnO nanorods, carbon black/PDMS	70%	30	0–45%, 45–70%	1.2 s	[43]
ZnSnO_3_ nanocubes and Ag-NWs	100%	−1	0–100%	-	[44]
Ag nanoparticles and CNTs/PDMS	95.6%	2.1	0–50%, 50–70%	-	[45]
Fe NWs@Graphene PUS	100%	14.5	0–100%	113 ms (10%)	This work

## Data Availability

The original contributions presented in this study are included in the article. Further inquiries can be directed to the corresponding authors.
